# Effectiveness of different treatment regimens on patients with COVID-19, hospitalized in Sanandaj hospitals: a retrospective cohort study

**DOI:** 10.1186/s40545-023-00511-w

**Published:** 2023-01-16

**Authors:** Jalal Asadi, Mohammad Aziz Rasouli, Ebrahim Ghaderi, Daem Roshani, Behzad Mohsenpour, Yousef Moradi, Ghobad Moradi

**Affiliations:** 1 Department of Epidemiology and Biostatistics, School of Medicine, Kurdistan University of Medical Sciences, Sanandaj, Iran; 2 Clinical Research Development Unit, Kowsar Hospital, Kurdistan University of Medical Sciences, Sanandaj, Iran; 3 Social Determinants of Health Research Center, Research Institute for Health Development, Kurdistan University of Medical Sciences, Sanandaj, Iran; 4 Department of Infectious Diseases, School of Medicine, Kurdistan University of Medical Sciences, Sanandaj, Iran

**Keywords:** COVID-19, Drug regimen, Treatment, Retrospective cohort, Iran

## Abstract

**Objectives:**

Different drugs have different effects on the prognosis of patients with COVID-19. This study aimed to evaluate the effect of different drug regimens on patients with COVID-19, hospitalized in Sanandaj city.

**Methods:**

In this retrospective cohort study, 660 patients with COVID-19, hospitalized in the Tohid, Kowsar and Besat hospitals located Sanandaj (Kurdistan Province, Iran) were studied from February 2020 to February 2021 with clinical symptoms and positive test results.

**Results:**

The results of multivariate regression analysis showed the days of hospitalization for patients who had received the drug regimen 2 (Interferons (ReciGen/Ziphron) or Interferon Vectra (lopinavir/ritonavir)) was 1.92 times higher than those who had received the drug regimen 1 (hydroxychloroquine group or a combination of chloroquine and azithromycin) while a significant association was observed (OR = 1.92, 95% CI: 1.16–3.16, *P* = 0.011). Also, the hospitalization in ICU was longer in patients treated by the drug regimen 2 (Interferons (ReciGen/Ziphron) or Interferon Vectra (lopinavir/ritonavir)) (OR = 4.63, 95% CI: 1.80–11.82, *P* = 0.001), however, drug regimens did not show a significant effect on mortality and use of ventilator in patients (*P* > 0.05).

**Conclusion:**

The study results showed the drug regimens 2 and 5 increased the days of hospitalization and hospitalization in ICU, respectively, while the other drug regimens had no significant effect on mortality and use a ventilator in the studied patients and none of the drug regimens had an effect on reducing mortality compared to other ones.

## Background

The first case of COVID-19 was observed in a seafood market in Wuhan, Hubei province, China on December 12, 2019, and the first positive case was diagnosed 21 days later, on January 3 in the United States [1, 2]. The very high outbreak potential led to the announcement of the COVID-19 global pandemic on March 11, 2020 [3]. The full range of COVID-19 disease is from a mild and self-limiting respiratory disease to severe progressive pneumonia, multiple organ failure, and death [4].

As of March 3, 2022, the total number of COVID-19 cases in the world were more than 438 million people and the number of deaths was over 5.960.000 patients. In the same period, the number of cases in the United States was 78 million people of whom 943.000 died. In Europe, the number of cases was 179 million people of whom 1,876,000 died. The number of positive cases in Africa was 8,500,000 people and the number of deaths was 169.000 [5]. In Iran, as of March 3, 2022, the number of positive cases was 7 million of whom 137,000 people died [6]. In Kurdistan province, from the beginning of the corona epidemic until January 24, 2022, about 41,000 positive cases were diagnosed and 1990 deaths occurred in the population of 1.5 million people of this province.

Mortality is associated with aging, the presence of comorbidities, greater severity of the disease, worsening of respiratory failure, high levels of D-dimer and C-reactive protein, a low number of lymphocytes and infections [7]. A retrospective cohort study in New York found out of 678 hospitalized patients with COVID-19, those with a high viral load had a higher independent mortality rate [8]. Preliminary published data show 25.9% of patients with COVID-19 need to be hospitalized in the ICU and 20.1% have the acute respiratory distress syndrome [9]. The mortality rate of COVID-19 in patients hospitalized in China, Italy, Spain and France was reported to be 4%, 13%, 11% and 15%, respectively [10], and according to the WHO, patients’ mortality rate was estimated at about 98.6% [11].

The rapid spread of infection worldwide has led to an urgent need for vaccines or therapeutic interventions to prevent or treat the disease [12]. Some results have shown drugs such as hydroxychloroquine, dexamethasone, *tocilizumab*, antiviral drugs (remdesivir, and favipiravir) have positive therapeutic effects [13]. Chloroquine phosphate was the first drug to be used in early clinical trials in China, and its efficacy against COVID-19 was reported [2]. A retrospective study in the US state of Virginia found no significant reduction in mortality or the need for mechanical ventilation was detected in patients admitted with COVID-19, treated by hydroxychloroquine with or without azithromycin [14]. Remdesivir is considered to be the most promising antiviral agent, which works by inhibiting the activity of RNA (RdRp)-dependent RNA polymerase. Favipiravir, another inhibitor of RdRp, or the anti-influenza agent has also been clinically evaluated for its effectiveness on COVID-19 patients [3]. The results of a systematic review evaluating the effects of remdesivir compared with placebo or standard care alone on clinical outcomes in hospitalized patients with COVID-19 showed remdesivir had no effect on their mortality until the 28th day of hospitalization and it made a small difference in patients’ mortality for any reason up to the 28th day after their infection. In addition, this drug had little effect on the independence of critically ill patients on ventilators [15]. Contradictory information due to the unknown quality of the disease caused the treatment process and instructions to be various in different countries so that the results of a study in France showed treatment with hydroxychloroquine was associated with a reduction in or disappearance of the viral load in COVID-19 patients and its effect was amplified by azithromycin [2].

Since COVID-19 became an epidemic, the effects of various antiviral drugs on patients with COVID-19 have been studied [16]. At present, patients with positive COVID-19 are receiving antiviral, antibiotic and steroid therapies [17]. The effect of these drugs on the final outcome of COVID-19 treatment is not yet clear, and it is possible that these drugs treat the patient and reduce the recovery time. Such information provides important opportunities and hopes for patients. In addition, it may give physicians the opportunity to use drugs associated with positive clinical outcomes in current COVID-19 management protocols [18].

Different drugs have different effects on patients with COVID-19. Therefore, considering the importance of this issue and choosing appropriate treatment and the fact that each of the above drugs have been used independently or with other drugs as drug regimens, we aimed to investigate the effectiveness of different treatment regimens in hospitalized patients with COVID-19.

## Methods

In this retrospective cohort study, 660 patients with Covid-19 were randomly selected based on the proportion of patients admitted to Tohid, Kowsar and Besat hospitals in Sanandaj (Kurdistan Province, Iran) in the period of February 2020 to February 2021 based on their positive Polymerase chain reaction (PCR) test. Patients’ information was collected from several different sources. First, information of patients related to each province and hospital was extracted from the portal of the Infectious Diseases Management Center of the Ministry of Health, Treatment and Medical Education of Iran, and in the second stage, it was extracted from the Hospital Information System. Hospital Information System (HIS) is a comprehensive and integrated information system designed to manage the administrative, financial and clinical aspects of a hospital [19]. In the third stage, this information was extracted from the data set of the hospital (MCMC) under the auspices of Kurdistan University of Medical Sciences. Finally, patients' additional information was collected from their medical records.

Demographic information (age, sex, place of residence, and occupation), clinical symptoms of the disease (fever, body aches, cough, sore throat, headache, shortness of breath, and diarrhea), laboratory information (WBC, Cr, BUN, and ESR), and underlying diseases (Cardiovascular diseases (CVD), chronic lung diseases, chronic kidney diseases (CKD), hypertension, asthma, chronic obstructive pulmonary diseases (COPD), diabetes, immunodeficiency and cancer were extracted and recorded from these electronic systems. Other patient information including the date of admission, the duration of hospitalization in the ICU, ventilator use, and the disease outcome (recovery or death) were recorded from these systems or through follow-up by researchers. The diagnosis confirmation was by reverse transcriptase polymerase chain reaction (RT-PCR) from two samples of oral and nasopharynx swab.

Drug regimens prescribed for patients during their hospitalization also included 6 groups according to the instructions of Iran, including: 1. hydroxychloroquine or a combination of chloroquine and azithromycin; 2. interferons (ReciGen/Ziphron) or interferon + Kaletra (lupinavir/ritonavir); 3. atazanavir; 4. remdesivir; 5. favipiravir; 6. corticosteroids (dexamethasone/methylprednisolone). The dose and duration of each drug regimen were collected from patients' medical records and final follow-up of patients was performed from the time of hospitalization until their discharge from the hospitals or death.

Baseline characteristics including demographic, and clinical information and laboratory results were estimated considering the different drug regimens. Univariate and multivariate regression analysis were used to evaluate the association between the final disease outcome (recovery or death), and the studied variables, including the days of hospitalization and hospitalization in ICU and the ventilator use according to the different drug regimens. In univariate analysis, variables with P value less than 0.2 (*P* < 0.2) were included in the final adjusted regression model and the odds ratio (OR) was estimated for each drug regimen.

The 5-, 10-, 15-, 20- and 30-day survival rate and median of survival were examined based on the variables under study. The difference in survival rate was measured for the subgroups using log-rank test. Using Kaplan–Meier method, overall survival, and type of treatment were demonstrated on a curve. All statistical analyses were performed using SPSS 24 and Stata16.0 software (StataCorp, College Station, TX).

## Results

Patients’ demographic, clinical and laboratory information according to different drug regimens are shown in Table 1. The results of the study showed the frequency of the days of hospitalization for more than 6 days was the highest in patients treated by the drug regimen 3 and the lowest in those receiving the drug regimen 2 while the frequency of the ICU admission was the highest in patients with the drug regimen 5. The use of a ventilator was higher in patients with the drug regimen 5 and the death outcome in patients receiving the drug regimen 5 and 6 was 29.8% and 21.5%, respectively (Table 1).

**Table 1:** Demographic and clinical characteristics of COVID-19 patients

Characteristic category	Total	Regim 1 *N* (%)	Regim 2 *N* (%)	Regim 3 *N* (%)	Regim 4 *N* (%)	Regim 5 *N* (%)	Regim 6 *N* (%)
Age group (years)							
≤ 65 years	391 (59.24)	53 (61.63)	139 (59.66)	27 (62.79)	47 (58.75)	25 (53.19)	100 (58.48)
> 65 years	269 (40.76)	33 (38.37)	94 (40.34)	16 (37.21)	33 (41.25)	22 (46.81)	71 (41.52)
Sex							
Male	365 (55.3)	47 (54.7)	139 (57.9)	28 (65.1)	46 (57.5)	46 (53.2)	84 (49.1)
Female	295 (47.7)	39 (45.3)	94 (42.1)	15 (34.9)	34(42.5)	46 (46.8)	87 (50.9)
Occupation							
Unemployed	202 (30.5)	8 (9.3)	10 (4.3)	4 (9.3)	4 (5)	0 (0)	10 (5.8)
Free	36 (5.5)	34 (39.5)	63 (27)	15 (34.9)	27 (33.8)	10 (21.3)	53 (31)
Retired	112 (17)	7 (8.1)	50 (21.5)	7 (16.3)	14 (17.5)	13 (27.7)	21 (3)
Housewife	265 (40.2)	34 (39.5)	92 (39.5)	15 (34.9)	28 (35)	18 (38.3)	78 (45.6)
Other	45 (6.8)	3 (3.5)	18 (7.7)	2 (3)	7 (8.8)	6 (12.8)	9 (5.3)
Days of hospitalization (day)							
≤ 6	313 (47.42)	49 (56.98)	95 (40.77)	24 (55.81)	37 (46.25)	24 (51.06)	84 (49.12)
> 6	347 (52.58)	37 (43.02)	138 (59.23)	19 (44.19)	43 (53.75)	23 (48.94)	87 (50.88)
Hospitalization in the ICU							
No	558 (84.5)	74 (86)	207 (88.8)	35(81.4)	64 (80)	31 (66)	147 (86)
Yes	102 (15.5)	12 (14)	26 (11.2)	8(18.6)	16 (20)	16 (34)	24 (14)
Use a ventilator							
No	619 (93.8)	80 (93)	244 (96.1)	37(86)	76 (95)	43(91.5)	159 (93)
Yes	48 (6.2)	6 (7)	9 (3.9)	6 (14)	4 (5)	4 (8.5)	12 (7)
Outcome							
Release	523 (79.2)	69 (80.2)	184 (179)	34 (79.1)	69 (86.2)	33 (70.2)	134 (78.4)
Death	137 (22.8)	17 (19.8)	49 (21)	9 (20.9)	11 (13.8)	14 (29.8)	37 (21.6)
Mean ± SD							
SPO2	90.23 ± 5.89	91.1 ± 4.06	90.43/±4.37	90.48 ± 4.39	90.3 ± 7.120	87.42 ± 11.34	90.21 ± 5.87
WBC	7.16 ± 4.15	7 ± 4.39	7.1 ± 2 ± 4.16	4.96 ± 7.23	4.25 ± 7.15	3.90 ± 7.89	7.07 ± 3.85
Cr	1.32 ± .98	1.12 ± 1.30	1.38 ± 1.08	1.26 ± .97	1.23 ± 0.74	1.26 ± 0.62	0.95 ± 1.37
BUN	20.57 ± 17.5	20.66 ± 14.65	20.14 ± 16.7	10.01 ± 17.23	22.45 ± 22.15	19.97 ± 10.29	18.39121.45 ±
ESR	32.42 ± 24.84	28.26 ± 2	24.13 ± 33.07	30.76 ± 26.74	24.13 ± 33.53	35.70 ± 27.67	32.56 ± 25.12
Cardiovascular disease							
No	561 (85)	56 (65.1)	204 (87.6)	40 (93)	74 (92.5)	44 (93.6)	143 (83.6)
Yes	99 (15	30 (34.9)	29 (12.4)	3 (7)	6 (7.5)	3 (6.4)	28 (4.7)
Diabetes							
No	581 (88.03)	74 (86)	195 (83.7)	42 (97.7)	73 (91.2)	42 (89.4)	155 (90.6)
Yes	79 (11.97)	12 (14)	38 (16.3)	1 (2.3)	7 (8.8)	5 (10.6)	16 (9.4)
Kidney disease							
No	649 (98.33)	85 (98.8)	227 (97.4)	43 (100)	80 (100)	46 (97.9)	168 (98.2)
Yes	11 (1.67)	1 (1.2)	6 (2.6)	0 (0)	0 (0)	1(2.1)	3 (1.8)
Liver disease							
No	655 (99.24)	86 (100)	231 (99.1)	43 (100)	80 (100)	46 (97.9)	169 (98.8)
Yes	5 (0.76)	0 (0)	2 (0.09)	0 (0)	0 (0)	1 (2.1)	2 (1.2)
Malignancy							
No	648 (98.18)	84 (97.7)	230 (98.7)	42 (97.7)	80 (100)	46 (97.9)	166 (97.1)
Yes	12 (1.82)	2 (2.3)	3 (1.3)	1 (2.3)	0 (0)	1 (2.1)	5 (2.9)
Immunodeficiency							
No	650(98.48)	86(100)	229 (98.2)	43 (100)	78 (97.5)	45 (97.3)	169 (98.8)
Yes	10 (1.52)	0 (0)	4 (1.7)	0 (0)	3 (2.5)	2 (4.3)	2 (1.3)
Lung disease							
No	639 (96.82)	82 (95.3)	227 (97.4)	40 (93)	79 (98.7)	45 (95.7)	166 (97.1)
Yes	21 (3.18)	4 (4.7)	6 (2.6)	3 (7)	1 (1.3)	2 (4.3)	5 (2.9)
Hypertension							
No	561 (85)	6 6(76.7)	197 (84.5)	37 (86)	69 (86.3)	42 (89.4)	150 (87.7)
Yes	99 (15)	20 (23.3)	36 (15.1)	6 (14)	11 (13.7)	5 (10.6)	21 (12.3)
Fever							
No	601 (91.06)	75 (87.2)	221 (94.8)	86) 37)	72 (9)	38 (80.1)	158 (92.4)
Yes	59 (8.94)	11 (12.8)	12 (5.2)	14) 6)	8 (10)	9 (19.1)	75 (87.2)
Body pain							
No	296 (44.85	66 (76.7)	91 (39.1)	30 (69.8)	27 (33.8)	14 (29.8)	85 (49.7)
Yes	364 (55.15)	20 (23.3)	142 (60.9)	13 (30.2)	53 (65.2)	33 (70.2)	86 (50.3)
Cough							
No	291 (44.09)	34 (39.5)	106 (43.5)	14 (32.6)	27 (33.7)	22 (46.8)	88 (51.5)
Yes	369 (55.91)	52 (60.5)	127 (54.5)	29 (67.4)	53 (66.3)	25 (53.2)	83 (48.5)
Diarrhea							
No	628 (95.15)	82 (95.3)	224 (96.1)	41 (95.3)	73 (91.3)	46 (97.9)	162 (86)
Yes	32 (4.85)	4 (4.7)	9 (3.9)	2 (4.7)	7 (8.7)	1 (2.1)	9 (16)
Shortness of breath							
No	221 (33.48)	34 (39.5)	70 (30)	8 (19.6)	22 (27.5)	20 (42.6)	67 (39.2)
Yes	439 (66.52)	52 (60.5)	163(70)	35 (81.4)	58 (72.5)	27 (53.86)	104 (60.8)
Headache							
No	516 (78.18)	72 (83.7)	184 (79)	30 (69.8)	64 (80)	34 (72.3)	132 (77.2)
Yes	144 (21.82)	14 (16.3)	49 (21)	13 (30.2)	16 (20)	13 (27.3)	39 (22.8)
Sore throat							
No	580 (87.88)	80 (93)	210 (90.1)	37 (93.5)	70 (87.5)	41 (87.2)	142 (83)
Yes	80 (12.2)	6 (7)	23 (9.9)	6 (6.5)	10 (12.5)	6 (12.8)	29 (17)

The results of multivariate regression analysis showed the odds of the days of hospitalization in patients who had received the drug regimen 2 was 1.92 times higher than those with drug regimen 1 and a significant correlation was observed (OR = 1.92, 95% CI: 1.16–3.16, *P* = 0.011) but there was no statistically significant association between other drug regimens and days of hospitalization. Also, the mortality rate did not show a significant association with the different drug regimens (*P* > 0.05) and there was no statistically significant association between the use of ventilators and the different drug regimens. The results of multivariate regression analysis showed people who had received the drug regimen 5 had a higher rate of hospitalization in ICU and the correlation was significant (OR = 4.63, 95% CI: 1.81–11.82, *P* = 0.001). However, there was no statistically significant association between the use of other drug regimens and the hospitalization in ICU (*P* > 0.05) (Table 2).

**Table 2:** Univariate and multivariate regression analysis for treatment of COVID-19

Characteristic category	Univariate analysis OR (95% CI)	*P-value	Multivariate analysis OR (95% CI)	*P*-value
Days of hospitalization				
Age group				
≤ 65 years	1 (Ref)		1 (Ref)	1(Ref)
> 65 years	1.12 (0.82–1.53)	0.468	1.12 (0.81–1.53)	0.479
Sex				
Female	1 (Ref)		Not in model	–
Male	0.78 (0.57–1.06)	0.121	–	–
Job				
Unemployed	1 (Ref)		Not in model	–
Self-employment	1.09 (0.53–2.22)	0.801	–	–
Retired	1.04 (0.49–2.20)	0.917	–	–
Housewife	1.52 (0.75–3.06)	0.235	–	–
Other	1.16 (0.48–2.80)	0.728	–	–
Cardiovascular	0.71 (0.46–1.09)	0.125	Not in model	–
Diabetes	1.37 (0.85–2.21)	0.191	Not in model	–
Kidney disease	1.59 (0.46– 5.48)	0.463	Not in model	–
Hypertension	0.99 (0.65–1.53)	0.991	Not in model	–
Drug regimen				
Regim 1	1 (Ref)		1 (Ref)	–
Regim 2	1.92 (1.16–3.17)	0.010	1.92 (1.16–3.16)	0.011
Regim 3	1.04 (0.50–2.19)	0.900	1.04 (0.50–2.19)	0.897
Regim 4	1.53 (0.83–2.84)	0.168	1.53 (0.83–2.83)	0.171
Regim 5	1.26 (0.62–2.59)	0.513	1.25 (0.61–2.56)	0.530
Regim 6	1.37 (0.81–2.31)	0.235	1.36 (0.81–2.30)	0.240
Outcome				
Age group				
≤ 65 years	1 (Ref)		1 (Ref)	–
> 65 years	3.61 (2.43–5.38)	< 0.001	3.06 (2.02– 4.63)	< 0.001
Sex				
Female	1 (Ref)		Not in model	–
Male	0.92 (0.63–1.34)	0.669	–	–
Job				
Unemployed	1(Ref)		Not in model	–
Self-employment	0.58 (0.25–1.35)	0.213	–	–
Retired	1.54 (0.65–3.60)	0.319	–	–
Housewife	0.69 (0.30–1.57)	0.386	–	–
Other	0.46 (0.14–1.44)	0.185	–	–
Cardiovascular	3.10 (1.96–4.91)	< 0.001	2.50 (1.51–4.13)	< 0.001
Diabetes	1.15 (0.65–2.03)	0.610	Not in model	
Kidney disease	1.59 (0.46–5.48)	0.463	Not in model	
Hypertension	1.86 (1.15–3)	0.011	1.32 (0.79–2.22)	0.281
Drug regimen				
Regim 1	1 (Ref)		1 (Ref)	
Regim 2	1.08 (0.5v8.2)	0.805	1.40 (0.71–2.75)	0.321
Regim 3	1.07 (0.43–2.65)	0.877	1.57 (0.59.415)	0.361
Regim 4	0.64 (0.28–1.48)	0.303	0.84 (0.34–2.05)	0.712
Regim 5	1.55 (0.67–3.56)	0.300	2.12 (0.85.5.24)	0.103
Regim 6	1.12 (0.58–2.13)	0.729	1.40 (0.70–2.82)	0.336
Use a ventilator				
Age group				
≤ 65 years	1 (Ref)	–	1 (Ref)	–
> 65 years	3.04(1.54–5.84)	0.001	2.41 (1.20–4.87)	0.013
Sex				
Female	1 (Ref)		Not in model	–
Male	0.93 (0.49–1.76)	0.827	–	–
Job				
Unemployed	1 (Ref)	-	Not in model	-
Self-employment	0.80 (0.30–2.10)	0.658	–	–
Retired	1.51 (0.56–4.02)	0.409	–	–
Housewife	0.78 (0.30–2.02)	0.617	–	–
Other	0.76 (0.22–2.62)	0.675	–	–
Cardiovascular	2.21 (1.06–4.57)	0.032	1.68 (0.76–3.73)	0.199
Diabetes	0.36 (0.85–1.52)	0.166	Not in Model	–
Kidney disease	1.52 (0.19–12.19)	0.692	Not in model	–
Hypertension	3.25 (1.64–6.46)	0.001	2.69 (1.30–5.54)	0.007
Drug regimen				
Regim 1	1 (Ref)	–	1 (Ref)	–
Regim 2	0.53 (0.18–1.55)	0.250	0.65 (0.21–2.00)	0.463
Regim 3	2.16 (0.65–7.15)	0.207	3.20 (0.88–11.55)	0.075
Regim 4	0.70 (0.19–2.58)	0.594	0.92 (0.23–3.63)	0.916
Regim 5	1.24 (0.33–4.63)	0.749	1.72 (0.42–6.96)	0.444
Regim 6	1 (0.36–2.77)	0.990	1.29 (0.44–3.80)	0.634
Hospitalization in the ICU				
Age group				
≤ 65 years	1 (Ref)	–	1 (Ref)	–
> 65 years	3.20 (2.06–4.98)	< 0.001	2.67 (1.67–4.25)	< 0.001
Sex				
Female	1 (Ref)	–	Not in model	–
Male	0.89 (0.58–1.36)	0.602	–	–
Job				
Unemployed	1 (Ref)	–	Not in model	–
Self-employment	0.45 (0.11–1.79)	0.261	–	–
Retired	1.32 (0.35–4.96)	0.681	–	–
Housewife	0.70 (0.19–2.55)	0.597	–	–
Other	0.51 (0.08–3.24)	0.477	–	–
Cardiovascular	2.43 (1.46–4.02)	0.001	2.17 (1.23–3.82)	0.007
Diabetes	0.58 (0.27–1.25)	0.167	Not in model	–
Kidney disease	1.22 (0.25–5.73)	0.801	Not in model	–
Hypertension	2.12 (1.27–3.54)	0.004	1.72 (0.99–2.98)	0.052
Drug regimen				
Regim 1	1 (Ref)	–	1 (Ref)	–
Regim 2	0.77 (0.37–1.61)	0.49	0.97 (0.44–2.13)	0.950
Regim 3	1.40 (0.52–3.75)	0.49	2.07 (0.73–5.90)	0.170
Regim 4	1.54 (0.67–3.49)	0.30	2.15 (0.88–5.22)	0.090
Regim 5	3.18 (1.34–7.50)	0.001	4.63 (1.80–11.82)	0.001
Regim 6	1.00 (0.47–2.12)	0.98	1.27 (0.57–2.81)	0.556

**Fig. 1:**
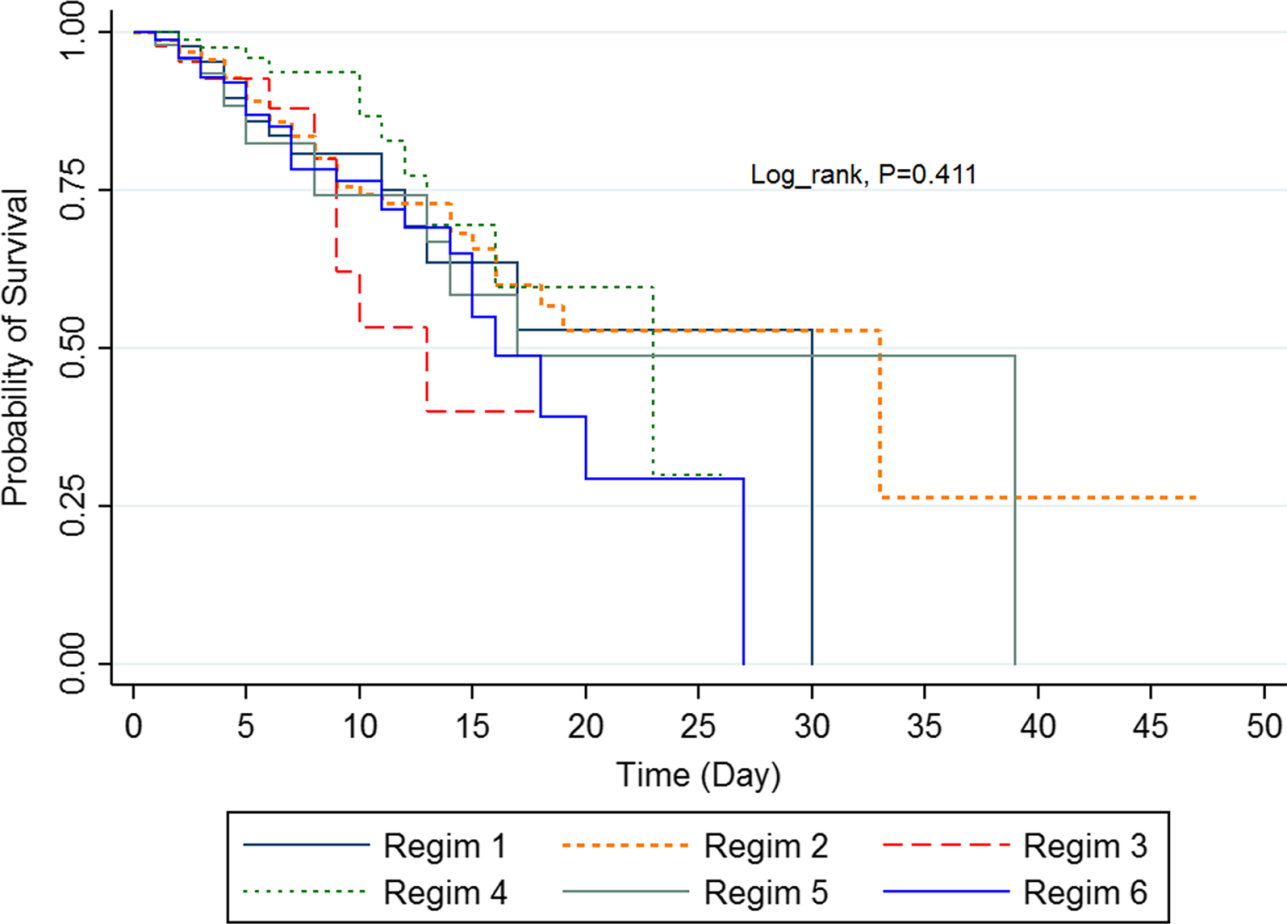
Kaplan–Meier curves of prostate cancer-specific survival across type of drug regimen

Patients’ overall survival rates at 5, 10, 15, 20 and 30 days were 88%, 74.4%, 59.4%, 45.5% and 28.9%, respectively. Also, patients’ survival probability curve based on the different drug regimens is shown in Fig. 1. The results of multivariate analysis of Cox regression showed the different drug regimens had no effect on the mortality rate of patients with Covid-19 (*P* > 0.05) (Table 3).

**Table 3:** Univariate and multivariate Cox regression model for mortality prediction

Characteristic category	Univariate analysis HR (95% CI)	*P-value	Multivariate analysis HR (95% CI)	*P*-value
Age group				
≤ 65 years	1 (Ref)		1 (Ref)	
> 65 years	2.42 (1.70–3.46)	< 0.001	1.83 (1.25–2.69)	0.002
Sex				
Female	1 (Ref)		Not in model	–
Male	0.92 (0.65–1.30)	0.658	–	–
Job				
Unemployed	1(Ref)		Not in model	–
Self-employment	0.72 (0.34–1.52)	0.395	–	–
Retired	1.25 (0.60–2.59)	0.319	–	–
Housewife	0.75 (0.36–1.52)	0.429	–	–
Other	0.61 (0.21–1.72)	0.354	–	–
Cardiovascular	2.67 (1.84–3.88)	< 0.001	1.89 (1.25–2.86)	0.002
Diabetes	1.03 (0.63–1.70)	0.881	Not in model	–
Kidney disease	0.41 (0.10–1.70)	0.223	Not in model	–
Hypertension	1.40 (0.93.2.10)	0.106	Not in model	–
Hospitalization in the ICU	4.39 (3.13–6.17)	< 0.001	3.68 (2.49–5.44)	< 0.001
Use a ventilator	3.13 (2.05–4.79)	< 0.001	1.59 (1.02–2.54)	0.048
Drug regimen				
Regim 1	1 (Ref)	–	1 (Ref)	
Regim 2	0.86 (0.49–1.49)	0.595	1.20 (0.67–2.16)	0.531
Regim 3	1.24 (0.55–2.80)	0.589	1.25 (0.54–2.92)	0.594
Regim 4	0.59 (0.27–1.26)	0.177	0.62 (0.27–1.40)	0.253
Regim 5	1.03 (0.49–2.14)	0.927	2.12 (0.30–1.58)	0.385
Regim 6	1.12 (0.63–2)	0.683	1.42 (0.77–2.62)	0.254

## Discussion

The results of multivariate regression analysis showed the hospitalization in ICU for people who had received the drug regimen 2 was 1.92 times higher than those receiving the drug regimen 1 and a significant correlation was observed. Also, the hospitalization in ICU was longer in those who had received the drug regimen 2, but the drug regimens did not show a significant effect on mortality and use of ventilators in the studied patients.

In a study conducted by Zhikang et al. on the efficacy and safety of corticosteroids on Covid-19 as a systematic review and meta-analysis, the results showed only corticosteroids were effective on reducing hospitalization days [20]. The results of another study by Lagie et al. in Marseille, France, on 3737 patients with Covid-19 on the hydroxychloroquine/azithromycin regimen and other regimens showed the mean hospitalization in ICU in the HCQ-AZ group was shorter than in other regimens (OR = 0.38, 95% CI: 0.27–0.54, *P* < 0.001), and HCQ-AZ consumption for more than 3 days was also an independent protective factor in transferring patients to the ICU [21].

In a retrospective study conducted by Vahedi et al. in Iran by comparing two different drug regimens, the results showed the antiviral drugs had no effect on the recovery of hospitalized patients and even led to an increase in their hospitalization duration [22].

In the present study, the results of multivariate regression analysis showed the different drug regimens had no significant effect on mortality of hospitalized patients (*P* > 0.05). These findings were consistent with those of two studies performed in the US, according to which hydroxychloroquine did not show an effect on mortality of hospitalized patients with Covid-19 [23, 24]. It should be noted that the use of antiviral drugs may be too late after symptoms occur in the patient and this explains their low efficiency in clinical settings.

The results of a retrospective cohort study by Arshada et al. showed treatment with hydroxychloroquine alone and the combination of two regimens of hydroxychloroquine and azithromycin were associated with a significant reduction (71% reduction) in mortality of hospitalized patients with Covid-19 [25].

The mortality rate associated with Covid-19 in the hospitals under study was 22.8%, one of the causes of which was the presence of comorbidities in Covid-19 patients hospitalized in these hospitals. Although the mortality rate was lower in the fourth group (remdesivir) than the other treatment groups, the results of multivariate regression showed there was no statistically significant correlation between the different drug regimens and mortality of hospitalized patients (*P* > 0.05).

In a study conducted by Garibaldi et al. in the United States in five hospitals to compare the clinical recovery time with remdesivir alone and remdesivir in combination with corticosteroids in patients with Covid-19, the results showed although the mortality rate was lower in the remdesivir group, it was not statistically significant [26]. This finding was consistent with the results of the present study. However, it should be noted that in studies performed on more than one treatment regimen and studies which are not of the clinical trial type, the random distribution of patients is not performed and the patient's condition determines the type of treatment, which affects the desired outcome.

In a study by Song Tong et al. in Wuhan, China, as a retrospective single center cohort study to compare mortality in patients treated with ribavirin for severe Covid-19, the results showed the mortality rate in the ribavirin group was 17.1% while in the untreated group, it was 24.6%, but there was no significant difference in the mortality between the two groups [27]. However, in various studies, analyses have shown timing is a key element in the treatment of this disease and it is clearly effective in reducing mortality in patients with Covid-19 [28]. Also, in determining the effect and difference of different treatment regimens on mortality of patients with Covid-19, age, sex and underlying diseases should be considered [29].

In the systematic review and meta-analysis conducted by Zhonga, the results showed treatment with lupinavir and ritonavir was not associated with clinical progression compared to standard care, but mortality after 28 days in the lupinavir and ritonavir group (1.5%) was 7% lower than that in the standard care group [30].

The results of systematic review and meta-analysis of Hosseinpour et al. to evaluate the efficacy and safety of favipiravir on the treatment of Covid-19 showed the mortality rate in the favipiravir group was approximately 30% lower than the control group, but this finding, consistent with the results of the present study, was not statistically significant (*P* = 0.95) [31].

In the present study, multivariate analysis using Cox regression modeling showed there was no significant association between the different drug regimens and the survival rate, which was in contrast to the study of Arshada et al. in the United States, the results of multivariate regression of which showed survival was higher in people treated by the hydroxychloroquine regimen alone and hydroxychloroquine in combination with azithromycin [25].

Another study was conducted by Somers in the United States on 154 patients to determine the effect of *tocilizumab* on the treatment of Covid-19 patients supported by a ventilator. Kaplan–Meier estimates in this study showed the survival probability was significantly higher in patients treated with *tocilizumab* compared to untreated patients (*P* = 0.0189) [32].

In our study, the results of multivariate regression analysis showed the hospitalization in ICU in people who had received the drug regimen 5 or favipiravir was 4.63 times higher than those treated by the drug regimen 1 or chloroquine and there was a significant correlation (OR = 3.46, 95% CI:1.8–11.82, *P* = 0.11) but there was no statistically significant association between the other drug regimens and hospitalization in ICU (*P* > 0.05). Multiple regression results of a retrospective study conducted in Turkey by Guner et al. with the aim of comparing the admission rate of Covid-19 patients treated with hydroxychloroquine, and a combination of favipiravir and hydroxychloroquine in intensive care units (ICU) showed there was no statistically significant difference between the HCQ group and the HCQ and favipiravir group in terms of ICU hospitalization, but compared to the HCQ group, the admission rate at ICU in the favipiravir group was significantly higher (OR = 9.70, 95% CI: 2–38.4) [33]. This finding was consistent with those of the present study.

In the study of Assiri et al. in Saudi Arabia, the results showed treatment with enoxaparin significantly reduced the hospitalization in ICU (*P* = 0.04) while the results showed the combined treatment with the three drugs lupinavir/ritonavir, ribavirin and interferon as well as tocilizumab led to an increase in patient hospitalization in ICU [18].

The results of the study of Fesharaki et al. in Iran showed that patients who were prescribed in the military hospital of antiviral drugs, antibiotic and corticosteroids compared to patients with nonmilitary hospital with similar treatment, have significantly lower ICU hospitalization [34].

In the present study, the results of multivariate regression analysis showed there was no statistically significant association between the different drug regimens and ventilator use (*P* > 0.05). In a study in the United States, no significant association was observed between different drug regimens and the need for mechanical ventilation in patients, which was consistent with the results of the present study [14]. The findings of another study in New York did not show a significant association between corticosteroids and mechanical ventilation in patients [35]. In a study conducted in Saudi Arabia, no significant association was observed between different drug regimens and the use of ventilators [36]. In another observational study conducted by Geleris, Joshua et al. at Columbia University on patients of New York, the results showed there was no significant association between patients who had received hydroxychloroquine and ones who had not receive the drug in the use of ventilators [24]. This finding was also consistent with the results of the present study.

## Conclusion

The results of this study showed the drug regimens 2 and 5 increased the days of hospitalization and hospitalization in ICU, respectively, and the other drug regimens had no significant effect on mortality and use of ventilators in the studied patients. None of the drug regimens had any effect on reducing mortality compared to other regimens. Clinical trials are suggested to be conducted based on different drug regimens at the appropriate time.

## Data Availability

The datasets used and analyzed during the current study are available from the corresponding author on reasonable request.

## Abbreviations

HIS: Hospital Information System
MCMC: Medical Care Monitoring Center
CKD: Chronic kidney diseases
COPD: Chronic obstructive pulmonary diseases
CVD: Cardiovascular diseases
